# Sustained seropositivity up to 20.5 months after COVID-19

**DOI:** 10.1186/s12916-022-02570-3

**Published:** 2022-10-13

**Authors:** Carlota Dobaño, Anna Ramírez-Morros, Selena Alonso, Rocío Rubio, Gemma Ruiz-Olalla, Josep Vidal-Alaball, Dídac Macià, Queralt Miró Catalina, Marta Vidal, Aina Fuster Casanovas, Esther Prados de la Torre, Diana Barrios, Alfons Jiménez, Jasmina Zanoncello, Natalia Rodrigo Melero, Carlo Carolis, Luis Izquierdo, Ruth Aguilar, Gemma Moncunill, Anna Ruiz-Comellas

**Affiliations:** 1 ISGlobal, Hospital Clínic - Universitat de Barcelona, Barcelona, Carrer Roselló 153 (CEK building), E-08036 Barcelona, Spain; 2 CIBER de Enfermedades Infecciosas, Barcelona, Spain; 3 Unitat de Suport a la Recerca de la Catalunya Central, Fundació Institut Universitari per a la recerca a l’Atenció Primària de Salut Jordi Gol i Gurina, Sant Fruitós de Bages, Spain; 4 Grup de Promoció de la Salut en l’Àmbit Rural (ProSaARu), Institut Català de la Salut, Sant Fruitós de Bages, Spain; 5 Facultat de Medicina, Universitat de Vic-Universitat Central de Catalunya (UVIC-UCC), Vic, Spain; 6 Biomolecular Screening and Protein Technologies Unit, Centre for Genomic Regulation (CRG), The Barcelona Institute of Science and Technology, Barcelona, Spain; 7 Centre d’Atenció Primària (CAP) Sant Joan de Vilatorrada. Gerència Territorial de la Catalunya Central, Institut Català de la Salut, Sant Fruitós de Bages, Spain

**Keywords:** COVID-19, SARS-CoV-2, Antibody, Seroprevalence, Kinetics, IgM, IgG, IgA, Health care workers, Duration

## Abstract

This study evaluated the persistence of IgM, IgA, and IgG to SARS-CoV-2 spike and nucleocapsid antigens up to 616 days since the onset of symptoms in a longitudinal cohort of 247 primary health care workers from Barcelona, Spain, followed up since the start of the pandemic. The study also assesses factors affecting antibody levels, including comorbidities and the responses to variants of concern as well as the frequency of reinfections. Despite a gradual and significant decline in antibody levels with time, seropositivity to five SARS-CoV-2 antigens combined was always higher than 90% over the whole study period. In a subset of 23 participants who had not yet been vaccinated by November 2021, seropositivity remained at 95.65% (47.83% IgM, 95.65% IgA, 95.65% IgG). IgG seropositivity against Alpha and Delta predominant variants was comparable to that against the Wuhan variant, while it was lower for Gamma and Beta (minority) variants and for IgA and IgM. Antibody levels at the time point closest to infection were associated with age, smoking, obesity, hospitalization, fever, anosmia/hypogeusia, chest pain, and hypertension in multivariable regression models. Up to 1 year later, just before the massive roll out of vaccination, antibody levels were associated with age, occupation, hospitalization, duration of symptoms, anosmia/hypogeusia, fever, and headache. In addition, tachycardia and cutaneous symptoms associated with slower antibody decay, and oxygen supply with faster antibody decay. Eight reinfections (3.23%) were detected in low responders, which is consistent with a sustained protective role for anti-spike naturally acquired antibodies. Stable persistence of IgG and IgA responses and cross-recognition of the predominant variants circulating in the 2020–2021 period indicate long-lasting and largely variant-transcending humoral immunity in the initial 20.5 months of the pandemic, in the absence of vaccination.

**Supplementary Information:**

The online version contains supplementary material available at 10.1186/s12916-022-02570-3.

## Introduction

The maintenance and effectiveness of adaptive immunity directed against SARS-CoV-2 after primary infection are key questions in understanding and controlling the COVID-19 pandemic and any future emerging new coronavirus threat. Despite the global start of vaccination campaigns by the end of 2020, a substantial percentage of the world’s population remains unvaccinated, and their capacity to resist infections relies only on naturally acquired immunity. We have previously shown that 90% of those infected with SARS-CoV-2 remain seropositive 1 year after discharge [1, 2]. To our knowledge, the duration of antibody responses following natural infection has not been assessed beyond 13-20 months to date [3–10].

SARS-CoV-2 elicits robust humoral immune responses, including production of virus-specific immunoglobulin M (IgM), IgA, and IgG. IgM and IgA isotypes dominate the early effector antibody response to SARS-CoV-2, and IgA greatly contributes to virus neutralization at mucosal sites [11, 12]. In serum, the three isotypes display neutralizing activity, with IgM and IgG1 (predominant subclass of IgG) being the most important contributors [13].

Reinfection and COVID-19 disease rates, including severe cases, may increase if immunity wanes in those who do not get vaccinated. The emergence of SARS-CoV-2 variants of concern (VoC) with high transmissibility and potentially lower susceptibility to antibodies has raised the question of whether antibodies induced by the original Wuhan strain will still protect against reinfections or only against severe COVID-19 [14]. Therefore, data on the long-term persistence and efficacy of the immune response is of vital importance to foresee the evolution of the COVID-19 pandemic especially with more contagious emerging variants like Delta and Omicron [15–18]. Data could also be useful to infer the potential duration of vaccine-elicited immunity, which started to be studied a year after the onset of the pandemic.

There is a wide heterogeneity in how individuals respond to SARS-CoV-2 infection in terms of type and potency of immune responses, resulting in diverse viral and clinical presentations and susceptibilities. Systematic reviews and meta-analyses have concluded that men, those over 65 years of age, smokers, and patients with comorbidities such as hypertension, diabetes, cardiovascular disease, cerebrovascular disease, chronic obstructive pulmonary disease (COPD), chronic kidney disease, and cancer, contribute significantly to disease severity and COVID-19 prognostic [19–27]. However, thus far, very few studies have assessed the effect of comorbidities on SARS-CoV-2 immune responses, including antibodies that mediate neutralizing protective effector functions [28, 29]. Furthermore, it is also likely that individuals also vary in their capacity to maintain protective antibody responses in time, and the factors determining humoral immune memory are not known.

The main objectives of this study were to evaluate the kinetics of anti-SARS-CoV-2 antibodies over a period of 20.5 months in convalescent unvaccinated individuals from a well-characterized longitudinal cohort of health care workers (HCW) (CoviCatCentral), to assess the effect of clinical and demographic variables on the antibody levels, and to estimate the prevalence of reinfections.

## Methods

### Study design and subjects

Two hundred forty-seven HCW presenting with COVID-19 in three primary care counties in Barcelona, Spain, were recruited in a prospective cohort from March 2020 [1] and followed up during 2021, with sample collection performed at different time points (T) per individual: T0, July–August 2020; T1, September 2020; T2, October 2020; T3, November 2020; T4, January-February 2021; T5, March–April 2021; T6, May–June 2021; T7, July 2021; and T8, November 2021. Infections were detected by antigen rapid diagnostic tests (RDTs) and quantitative reverse transcription polymerase chain reaction (RT-qPCR) performed on participants with symptoms of COVID-19 or who had been in close contact with someone with SARS-CoV-2 infection. Overall, primary infections occurred between pre-T0 and T4. The effect of baseline characteristics on the anti-SARS-CoV-2 antibody response 1 year after the onset of the pandemic has already been reported [1] except for comorbidities and other risk factors that are addressed here: chronic kidney disease, COPD, asthma, cardiovascular disease, neurological diseases, digestive diseases, autoimmune diseases, cancer, immunosuppression (disease or drug-related), obesity, pregnancy, diabetes mellitus, dyslipidemia, hypertension, depression and/or anxiety, and hypothyroidism. Anti-SARS-CoV-2 serologic testing was performed at nine cross-sectional visits, and data on those not being vaccinated by mid-November 2021 is analyzed here. The later visits included in this analysis were T6 (May-June 2021), T7 (July 2021), and T8 (November 2021). The baseline (T0, July–August 2020) sample was obtained from the SeroCatCentral/VisCat study. T6 (*N* = 72) included 22 physicians or dentists, 35 nurses, and 15 with other job categories like customer and social services staff, with median (IQR) age of 45 (13) years and 86.3% being women; T7 (*N* = 39) included 11 physicians/dentists, 21 nurses, and 7 others, with median (IQR) age of 48 (13) years and 87.2% women; T8 (*N* = 23) included 4 physicians/dentists, 13 nurses, and 6 others, with median (IQR) age of 49 (13) years and 87% women.

The study protocols were approved by the IRB *Comitè Ètic d’Investigació Clínica IDIAP Jordi Gol* (codes 20/186-PCV, 20/094-PCV and 20/162-PCV), and written informed consent was obtained from participants.

### SARS-CoV-2 antibody measurements

Naturally acquired IgM, IgA, and IgG responses to SARS-CoV-2 were quantified by Luminex. The antigen panel included five proteins: the spike full length protein (S) (aa 1-1213 expressed in Expi293 and His tag-purified) produced at the Center for Genomic Regulation (CRG, Barcelona), and its subregion S2 (purchased from SinoBiological), the receptor-binding domain (RBD) kindly donated by the Krammer lab (Mount Sinai, New York), the nucleocapsid (N) full length (FL) protein, and the specific C-terminal (CT) region (both expressed in-house in ISGlobal in *E. coli* and His tag-purified). In addition, the RBD proteins of four VoC (Alpha, Beta, Gamma and Delta, produced at CRG) were tested in the first and last three visits. Coupling of SARS-CoV-2 proteins to MagPlex® polystyrene 6.5 μm COOH-microspheres (Luminex Corp, Austin, TX, USA) was done as described [1, 30, 31]. Antigen-coupled microspheres were added to a 384-well Clear® flat bottom plate (Greiner Bio-One, Frickenhausen, Germany) in multiplex (2000 microspheres per analyte per well) in a volume of 90 μL of Luminex Buffer (1% BSA, 0.05% Tween 20, 0.05% sodium azide in PBS) using 384 channels Integra Viaflo semi-automatic device (96/384, 384 channel pipette). Two hyperimmune pools (one for IgG, and another one for IgA and IgM) were used as positive controls in each assay plate for QA/QC purposes and were prepared at 2-fold, 8 serial dilutions from 1:12.5. Pre-pandemic samples were used as negative controls to estimate the cutoff of seropositivity. Ten microliters of each dilution of the positive control, negative controls, and test samples (prediluted 1:50 in 96 round-bottom well plates) was added to a 384-well plate using Assist Plus Integra device with 12 channels Voyager pipette. Plasma samples had been previously assessed for optimal sample dilution to avoid saturated responses, tested here at 1:500. To quantify IgM and IgA responses, test samples and controls were pre-treated with anti-human IgG (Gullsorb) at 1:10 dilution, to avoid IgG interferences. Technical blanks consisting of Luminex Buffer and microspheres without samples were added in 4 wells to detect and adjust for non-specific microsphere signals. Plates were incubated for 1 h at room temperature in agitation (Titramax 1000) at 900 rpm and protected from light. Then, the plates were washed three times with 200 μL/well of PBS-T (0.05% Tween 20 in PBS), using BioTek 405 TS (384-well format). Twenty-five microliters of goat anti-human IgG phycoerythrin (PE) (GTIG-001, Moss Bio) diluted 1:400, goat anti-human IgA-PE (GTIA-001, Moss Bio) 1:200, or goat anti-human IgM-PE (GTIM-001, Moss Bio) 1:200 in Luminex Buffer was added to each well and incubated for 30 min. Plates were washed and microspheres resuspended with 80 μL of Luminex Buffer, covered with an adhesive film, and sonicated 20 s on sonicator bath platform, before acquisition on the Flexmap 3D® reader. At least 50 microspheres per analyte per well were acquired, and median fluorescence intensity (MFI) was reported for each analyte. Assay positivity cut-offs specific for each isotype and antigen were calculated as 10 to the mean plus 3 standard deviations of log_10_-transformed MFI values of 128 pre-pandemic controls (Additional file 1: Fig. S1). Positive serology was defined by being positive for IgG, IgA and/or IgM to any of the SARS-CoV-2 wild type of the antigens tested (NFL, NCT, S, RBD, S2).

### Data analysis

We modeled antibody level trajectories over time with linear mixed models (LMM) using linear and quadratic fix effect terms for the time since infection and a random effect intercept to account for the dependency of longitudinal observations coming from the same individual. We repeatedly fitted LMMs changing our outcome of interest, which were the log_10_(MFI) for the different antigen and antibody isotype pairs. Considering that we modeled the log_10_(MFI) and that MFI signal is supposed to be relatively linear with antibody levels, negative (or positive) linear trends imply a constant negative (or positive) exponential antibody levels decay (or growth), whereas deviations from a linear trend for the log_10_(MFI) imply an acceleration or deceleration of the exponential antibody change. Estimated fixed effect regression coefficients and their standard deviations were used for prediction of temporal curves of antibody population averages and their 95% confidence intervals (CI). The associations between baseline determinants, clinical presentations, comorbidities and levels of antibodies were assessed at the time point closest to infection (between 5 and 9 months) and, at a later time point just prior to vaccination, about a year after infection (T4). Both univariable linear regression and stepwise regression models were fit to determine the effects of baseline variables on antibody levels (log_10_MFI). Multivariable models were selected based on the Akaike and Bayesian information criteria and adjusted r-square parameter. Finally, the formulas of the models were selected specifically at the antibody isotype level. For an easier interpretation of the results, a transformed beta value (%) of the log-linear model was calculated with the formula: ([10^beta]-1)*100, giving the difference (in percentage) in antibody levels when comparing to the reference group for categorical variables or for a one-unit increase for continuous variables. Likewise, a transformed beta value (%) of the log-log model was calculated with the formula: ([10^(beta*log_10_(1.1))]-1)*100, giving the difference (in percentage) in antibody levels for a 10% increase of the predictor variable, for continuous variables. Finally, we also assessed the association of the same baseline variables with differences in the rate of antibody changes as were estimated in our LMM fits of each antibody isotype kinetics. This association was estimated as a fix effect interaction with the time since symptom onset and was repeatedly estimated for all variables while controlling for a false discovery rate of 5%. Reinfected individuals were not excluded from the analysis of antibody kinetics or from the models to assess the associations of variables with antibody levels or decay. *p*-values were considered statistically significant at the 5% level. All data collected were managed and analyzed using the R software version 4.1.2.

## Results and discussion

**Fig. 1:**
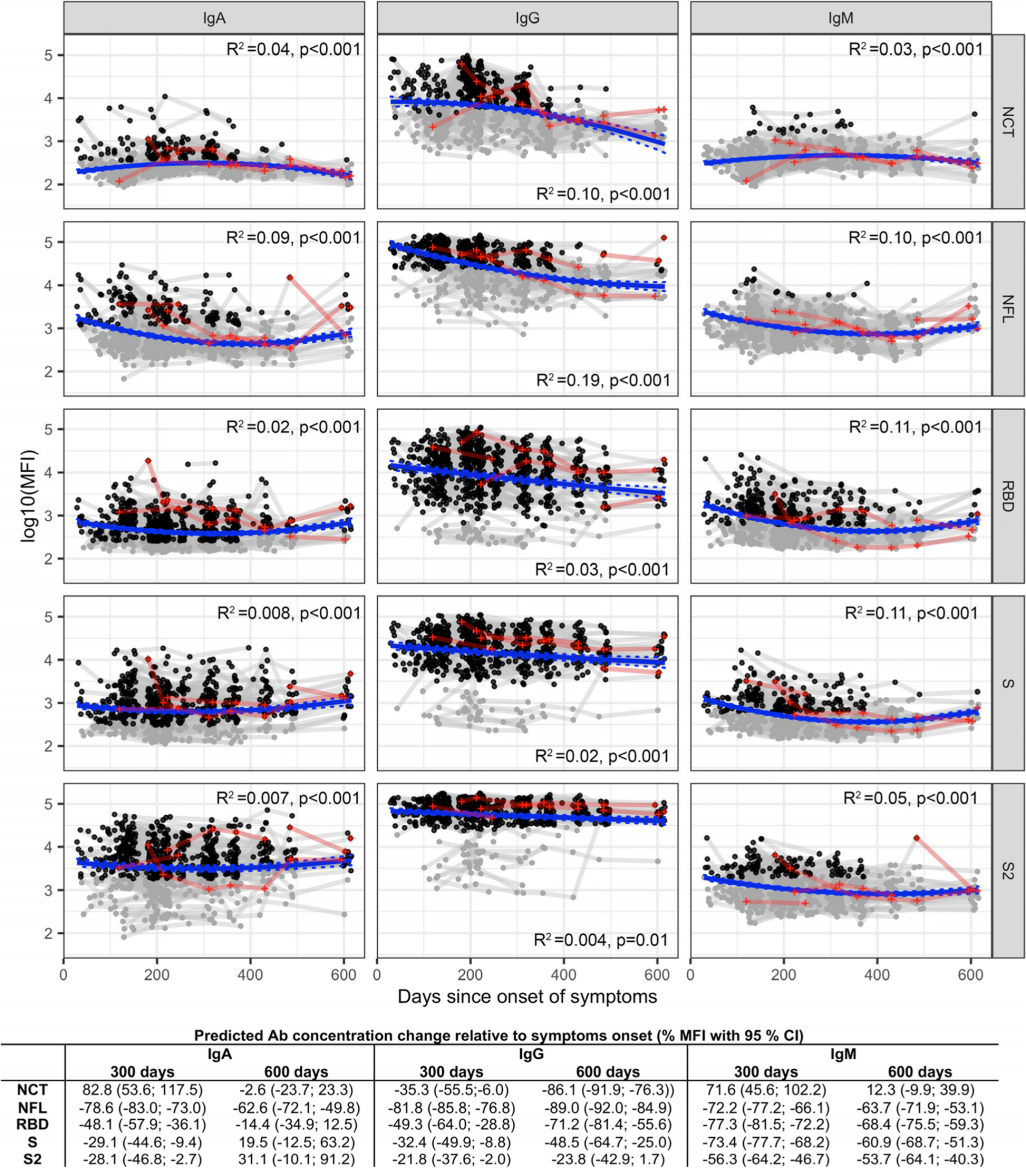
SARS-CoV-2 seropositivity in a cohort of pre-exposed non-vaccinated health care workers over 2020 and 2021. SARS-CoV-2 IgA, IgG, and IgM antibody (Ab) levels (log_10_ median fluorescence intensity, MFI) by days since COVID-19 symptoms onset. Black dots represent seropositive and gray ones represent seronegative responses. Samples from the same participant are joined by gray lines. Highlighted in red are samples from individuals after a documented reinfection by RT-qPCR. The blue solid line represents the predicted population average calculated using linear mixed models with linear and quadratic fix effect terms for the dependency on time since symptoms onset. Dashed lines correspond to 95% confidence interval. Predicted antibody level changes relative to levels at the onset of symptoms are reported in the table below at 300 and 600 days after it. Reported marginal *R*^2^ gives a measure of the goodness of fit and corresponds to the ratio of variance explained by time since infection over the total variance of the outcome, including the modeled random intercept. Significance of fits departing from that of lack of antibody change (null hypothesis) were assessed using a log-likelihood ratio test comparing a full model containing a linear and quadratic term for time since infection and a reduced model containing none of them. Ab, antibody

Of the total 247 HCW with past COVID-19 disease included in the cohort, Table 1 shows the number of non-vaccinated participants tested serologically per visit (T0–T8), involving 809 plasma samples and 15,267 antibody-antigen pair measurements overall. Among them, SARS-CoV-2 seropositivity combining all Ig isotypes and antigens was > 95% up to November 2021 (*N* = 23). The highest seropositivity was for IgG (~96%), especially for anti-S and anti-RBD responses, and IgA (~96%), mainly for anti-S responses. Seropositivity for IgM was ~48%, mainly for anti-RBD responses. Compared to July 2021, IgG levels remained stable and IgA and IgM seropositivity was increased in November 2021, probably due to an increase in asymptomatic infections, coinciding with the start of the sixth wave in Catalonia. This increase can be observed in the trajectory plots between T7 and T8 in Fig. 1, and it is especially evident for IgM to RBD and IgA to RBD and NFL. The kinetics of antibody levels up to 616 days since symptoms onset are shown in Fig. 1. The decay was more pronounced for anti-N than anti-S IgGs, with a remarkable sustain of S and S2 antibodies, less so for RBD. Overall, there was a slight but significant increase in IgA levels to S and S2 with time as observed by the predicted positive change in levels (Fig. 1), in contrast to the gradual decrease in antibody levels to the other antigens. Consistently, multivariable models at T4 had negative beta coefficients for all except IgA to S antigens that did not significantly wane with days since symptoms onset (Additional file 1: Table S1). Anti-S IgA unexpected rise might be related to sub-patent re-exposures resolved at the mucosal compartment. Thus, antibody kinetics after natural infection appeared to be more stably sustained than that after COVID-19 vaccination, which has been reported by vaccine manufacturers to decline more pronouncedly by 6–9 months [32–34].

**Table 1:** SARS-CoV-2 seropositivity (overall, by isotype, and by isotype-antigen pair) in a cohort of pre-exposed non-vaccinated health care workers over 2020 and 2021

	T0 (***n*** = 127)		T1 (***n*** = 122)		T2 (***n*** = 118)		T3 (***n*** = 52)		T4 (***n*** = 151)		T5 (***n*** = 105)		T6 (***n*** = 72)		T7 (***n*** = 39)		T8 (***n*** = 23)	
	***n***	%	***n***	%	***n***	%	***n***	%	***n***	%	***n***	%	***n***	%	***n***	%	***n***	%
**Overall seropositivity**	118	92.91%	111	90.98%	109	92.37%	49	94.23%	145	96.03%	105	100%	71	98.61%	38	97.44%	22	95.65%
**IgM seropositivity**	81	63.78%	65	53.28%	54	45.76%	22	42.31%	76	50%	48	45.71%	11	15.28%	4	10.26%	11	47.83%
IgM N CT	5	3.94%	3	2.46%	3	2.54%	3	5.77%	6	3.97%	2	1.90%	1	1.39%	0	0%	1	4.35%
IgM N FL	5	3.94%	2	1.64%	1	0.85%	0	0%	2	1.32%	1	0.95%	1	1.39%	0	0%	1	4.35%
IgM RBD	70	55.12%	54	44.26%	44	37.29%	16	30.77%	57	37.75%	41	39.05%	11	15.28%	3	7.69%	11	47.83%
IgM S	35	27.56%	38	31.15%	32	27.12%	16	30.77%	45	29.80%	28	26.67%	3	4.17%	1	2.56%	2	8.70%
IgM S2	22	17.32%	21	17.21%	20	16.95%	10	19.23%	34	22.52%	17	16.19%	1	1.39%	1	2.56%	0	0%
**IgA seropositivity**	112	88.19%	100	81.97%	90	76.27%	43	82.69%	134	88.74%	94	89.52%	57	79.17%	30	76.92%	22	95.65%
IgA N CT	12	9.45%	29	23.77%	24	20.34%	19	36.54%	56	37.09%	12	11.43%	2	2.78%	1	2.56%	1	4.35%
IgA N FL	33	25.98%	18	14.75%	23	19.49%	12	23.08%	37	25%	20	19.05%	4	5.56%	4	10.26%	8	34.78%
IgA RBD	82	64.57%	86	70.49%	75	63.56%	40	76.92%	114	75.50%	84	80.00%	32	44.44%	14	35.90%	17	73.91%
IgA S	105	82.68%	91	74.59%	80	67.80%	42	80.77%	115	76.16%	82	78.10%	52	72.22%	28	71.79%	22	95.65%
IgA S2	97	76.38%	73	59.84%	72	61.02%	40	76.92%	112	74.17%	78	74.29%	40	55.56%	19	48.72%	21	91.30%
**IgG seropositivity**	118	92.91%	110	90.16%	108	91.53%	47	90.38%	143	94.70%	104	99.05%	70	97.22%	38	97.44%	22	95.65%
IgG N CT	14	11.02%	100	81.97%	83	70.34%	29	55.77%	97	64.24%	25	23.81%	3	4.17%	2	5.13%	0	0%
IgG N FL	110	86.61%	98	80.33%	83	70.34%	23	44.23%	76	50.33%	49	46.67%	8	11.11%	5	12.82%	9	39.13%
IgG RBD	118	92.91%	110	90.16%	108	91.53%	47	90.38%	142	94.04%	104	99.05%	64	88.89%	35	89.74%	22	95.65%
IgG S	118	92.91%	109	89.34%	106	89.83%	47	90.38%	142	94.04%	102	97.14%	70	97.22%	38	97.44%	22	95.65%
IgG S2	118	92.91%	108	88.52%	104	88.14%	44	84.62%	141	93.38%	102	97.14%	66	91.67%	38	97.44%	22	95.65%

T, time point; T0, July–August 2020; T1, September 2020; T2, October 2020; T3, November 2020; T4, January–February 2021; T5, March–April 2021; T6, May–June 2021; T7, July 2021; T8, November 2021. Numbers in parentheses refer to samples tested

*n*, number of individuals who donated samples per time point (first row) and were positive by serology for each antibody/antigen pair (subsequent rows). Those who just received a first vaccine dose in the prior 6 days were included. The total number of pre-exposed individuals in the study cohort who were not vaccinated in 2021 was 161 at T4, 109 at T5, 77 at T6, 44 at T7, and 30 at T8. *N* nucleocapsid, *FL *full length, *CT *C-terminus, *S *spike, *RBD *receptor-binding domain

We repeated the longitudinal analysis excluding post-reinfection samples from the 8 participants for which we had RT-qPCR diagnosed reinfections and obtained nearly identical results. Some quadratic models gave a positive slope at long times since infection, which could be due to asymptomatic reinfections by Delta and/or Omicron variants, and/or poor goodness of fit for antibody levels owing to the sparsity of data at this interval of time and to the relative simplicity of the model we chose (quadratic) to avoid overfitting.

There was substantially lower binding of circulating antibodies to RBD Beta, followed by Gamma and Delta variants, compared to the wild type, and less difference for Alpha, with an increase in seroprevalence at the later time points (Additional file 1: Table S2). Alpha (B.1.1.7) was first detected in the study area in the summer of 2020 (when B.1.177 was the predominant lineage) [35] and prevailed from February (> 50%) till June 2021 (80–99% cases). Delta (B.1.617.2) was first detected in May 2021, raising to 10% in June, and predominating since July (> 50%) till November 2021 (80–100% cases). Omicron (B.1.529) was first detected early December and was already majoritarian (> 56%) in January 2022. Beta and Gamma frequencies were negligible. Thus, the raise in seropositivity against Delta by T8 could be a mixture of cross-recognition and undetected asymptomatic reinfections at the fifth Spanish pandemic wave (summer–fall 2020).

According to the USA Centers for Disease Control and Prevention [36], reinfection is defined as occurring ≥ 90 days after initial positive testing or ≥ 45 days with background information supporting contact with confirmed cases or the reappearance of COVID-19–like symptoms. In our high-risk population (frontline unvaccinated HCW), there were 8/247 reinfections (incidence of 3.23%), with a mean time between first and second infection of 279 days (range 58–586). In a meta-analysis of 19 studies [37], the incidence of reinfection in recovered COVID-19 patients ranged from 0 to 20%. The pooled reinfection rate was 0.65% (95% CI 0.39–0.98%), with high heterogeneity (*I*^2^ = 99%). One of the studies showing a higher incidence of reinfection (15%) was in HCW from a hospital in Barcelona [38]. In our cohort, the mean age of reinfected individuals was 43.9 ± 9.5 years, 7 were female, and 62.5% had a comorbidity. The comorbidities, clinical presentations, dates of infections, and serology are presented in Additional file 1: Table S3. Seven of the reinfections were symptomatic, 85.7% had similar clinical symptoms in both episodes, and 14.3% had a milder form of disease in the second episode. In no case was the second infection more severe than the first, in contrast to another study where 27.8% of reinfected patients had more severe symptoms in the second episode [39].

Before the second positive RT-qPCR diagnosis, five reinfection cases had negative serology, one was undetermined, and two had positive serology. Among the latter, one (asymptomatic) had a weak antibody positive response, and the other (reporting a close positive contact) had a strong serological response (RBD IgG 10 times above the cutoff, S IgG 8 times above the cutoff). In this second case, the reinfection was with Delta. According to the Public Health England report, Delta increased the chances of reinfection by up to 46% compared to Alpha [40]. Overall, serology data suggest that most of the reinfections were due to insufficient natural immunity [36, 41], and the last case was probably due to immune escape, i.e., naturally acquired immunity to the original variant was not effective against Delta [42, 43]. In this subset of individuals with reinfections, there were significant increases in the slope for IgG (RBD, *p* = 0.027; S, *p* = 0.008; S2, *p* = 0.014) and IgA (S, *p* = 0.023; S2, *p* = 0.014) levels, all with rho > 0.21.

Two hundred twenty-three patients (90%) had at least one comorbidity. The most frequent was depression/anxiety (19.3%), followed by having had previous allergies (15.7%) and dyslipidemia (14.8%). We assessed baseline factors and comorbidities associated to antibody levels measured in the first sample post-infection available from each participant (from 5 to 9 months post infection) by multivariable stepwise regression models adjusting by time since infection (Table 2). Baseline variables most consistently and significantly associated with higher antibody levels 5–9 months after infection were age, obesity (*n* = 24), hypertension (*n* = 18), and variables related to the initial COVID-19 episode: hospitalization (*n* = 25), fever (*n* = 163), anosmia and/or hypogeusia (*n* = 133), chest pain (*n* = 41), and duration of symptoms (Table 2). Specifically, age was positively associated with anti-N IgA and IgG responses, having 2–2.5% higher levels with each year older. Hypertensive individuals had 57% higher N FL IgA levels, and obesity was associated with 25% lower N FL IgM levels. HCW who had anosmia/hypogeusia or fever had significantly higher IgG levels to all antigens than those without these conditions. Chest pain was associated with 20% higher N CT IgM levels. Higher IgA was positively associated with symptoms duration (median 22 days, IQR 12–34; N FL, rho = 0.116, *p* = 0.083; RBD, rho = 0.238, *p* < 0.001; S, rho = 0.244, *p* < 0.001). Hospitalized patients had 79% times higher RBD IgA levels than those non-hospitalized. Baseline factors associated with lower IgG levels included smoking, with 44% less IgG to N CT, 36% less to N FL and 51% less to RBD than non-smokers (Table 2). Variables significantly associated with antibody levels ~1 year after infection and just before most HCW received the first vaccine dose (T4), are shown in Additional file 1: Table S1. Additional factors significantly associated with lower IgA and IgG levels later on were being physician or nurse compared to other occupations in the primary care health sector and headache symptoms during the initial COVID-19 episode. All other variables, symptoms, or sequelae were either not statistically significantly associated with antibody levels or weakly associated in univariable models. Apart from the reported associations with antibody levels at the time closest to and farthest from infection, we also assessed a potential association of the same variables with differences in the rate of antibody changes as estimated in Fig. 1. The most consistent significant variables were tachycardia and cutaneous symptoms, associated with slower antibody decay, and oxygen supply, with faster antibody decay (Additional file 1: Table S4).

**Table 2:** Factors affecting Ig levels (log_10_ median fluorescent intensity) 5–9 months after COVID-19 by multivariable stepwise regression models

		N CT	N FL	RBD	S	S2
		Beta (%) 95% CI	Beta (%) 95% CI	Beta (%) 95% CI	Beta (%) 95% CI	Beta (%) 95% CI
**IgA**	Age	**0.9 (0.15; 1.66)**	**1.9 (0.77; 3.04)**	0.48 (− 0.46; 1.43)	0.98 (− 0.23; 2.22)	1.02 (− 0.54; 2.61)
	Shivers	7.38 (− 8.68; 26.27)	17.13 (− 7.94; 49.02)	18 (− 3.79; 44.74)	18.04 (− 9.27; 53.59)	9.08 (− 22.22; 52.98)
	Symptoms duration	0.21 (− 0.09; 0.52)	**0.46 (0.01; 0.92)**	**0.48 (0.1; 0.87)**	**0.57 (0.07; 1.06)**	0.35 (− 0.29; 0.99)
	Sputum	− 0.53 (− 27.1; 35.73)	21.77 (− 23.28; 93.26)	26.52 (− 14.49; 87.19)	4.24 (− 37.08; 72.7)	− 12.9 (− 54.47; 66.64)
	Fever	− 5.07 (− 21; 14.07)	9.16 (− 16.92; 43.43)	13.39 (− 10.05; 42.93)	28 (− 5.02; 72.5)	26.53 (− 13.77; 85.65)
	Anosmia/hypogeusia	7.96 (− 7.85; 26.48)	− 1.43 (− 22.1; 24.72)	3.22 (− 15.45; 26.03)	24.88 (− 3.44; 61.51)	**49.2 (7.2; 107.64)**
	Hospitalization	14.35 (− 23.18; 70.2)	− 8.98 (− 49.6; 64.39)	**79.25 (8.58; 195.91)**	47.25 (− 22.82; 180.94)	85.13 (− 19.29; 324.64)
	Hypertension	18.89 (− 11.13; 59.07)	**56.55 (1.57; 141.29)**	8.63 (− 24.73; 56.77)	− 12.94 (− 45.74; 39.69)	12.92 (− 38.5; 107.32)
	Dizziness	− 4.2 (− 21.91; 17.53)	− 1.46 (− 27.28; 33.52)	18.82 (− 8.17; 53.73)	20.71 (− 13.39; 68.25)	35.75 (− 11.4; 107.98)
	Oxygen	17.09 (− 27.3; 88.57)	35.15 (− 33.44; 174.42)	− 18.38 (− 55.23; 48.81)	− 13.7 (− 60.2; 87.14)	− 34.56 (− 75.8; 76.93)
	Cough	9.41 (− 7.11; 28.87)	4.24 (− 18.27; 32.95)	6.83 (− 13.09; 31.31)	14.26 (− 12.42; 49.07)	3.92 (− 26.15; 46.25)
**IgG**	Age	**2.71 (1.37; 4.08)**	**2.23 (1.2; 3.27)**	**2.3 (0.66; 3.97)**	1.31 (− 0.2; 2.84)	0.69 (− 0.4; 1.79)
	Shivers	27.7 (− 5.25; 72.12)	12.11 (− 10.82; 40.94)	27.37 (− 11.63; 83.59)	17.65 (− 16.21; 65.21)	6.24 (− 16.88; 35.79)
	Dyspnea	8.58 (− 20.4; 48.09)	1.35 (− 20.11; 28.57)	− 0.57 (− 32.02; 45.42)	3.96 (− 26.96; 47.97)	− 4.24 (− 25.81; 23.59)
	Fever	**107.38 (49.19; 188.25)**	**89.46 (47.19; 143.88)**	**192.5 (95.41; 337.82)**	**152.84 (73.85; 267.7)**	**82.01 (38.84; 138.59)**
	Anosmia/hypogeusia	**50.43 (13.84; 98.8)**	**58.13 (27.7; 95.8)**	**90.52 (35.41; 168.06)**	**104.14 (48.67; 180.3)**	**73.92 (38.3; 118.71)**
	Hospitalization	36.15 (− 31.97; 172.47)	7.73 (− 36.71; 83.38)	52.13 (− 34.96; 255.88)	38.26 (− 37.19; 204.38)	29.59 (− 26.74; 129.24)
	Dizziness	4.2 (− 27.37; 49.48)	12.64 (− 14.58; 48.54)	17.46 (− 24.5; 82.75)	6.62 (− 29.27; 60.73)	3.68 (− 22.94; 39.49)
	Myalgia/arthralgia	− 0.88 (− 26.47; 33.62)	− 5.9 (− 25.16; 18.31)	− 16.5 (− 42.08; 20.39)	− 9.84 (− 35.81; 26.64)	− 8.22 (− 28.2; 17.32)
	Oxygen	13.09 (− 51.02; 161.11)	14.62 (− 39.65; 117.71)	38.62 (− 50.26; 286.33)	27.63 (− 50.73; 230.58)	6.18 (− 46.63; 111.25)
	Cough	23.2 (− 7.49; 64.07)	21 (− 2.86; 50.73)	29.93 (− 8.52; 84.56)	23.34 (− 10.96; 70.86)	21.7 (− 3.84; 54.03)
	Ex-smoker	0.69 (− 27.97; 40.75)	− 4.64 (− 26.23; 23.28)	4.65 (− 30.57; 57.72)	5.92 (− 27.63; 55.04)	2.3 (− 22.32; 34.72)
	Smoking	− 43.78*(− 66.34; − 6.09)	**− 36.12 (− 56.9; − 5.34)**	**− 51.11 (− 73.92; − 8.34)**	− 44.12 (− 68.82; 0.16)	− 32.05 (− 55.43; 3.61)
**IgM**	Shivers	− 6.18 (− 18.05; 7.4)	0.41 (− 15.79; 19.72)	14.29 (− 9.05; 43.62)	15.08 (− 5.92; 40.77)	7.81 (− 12.62; 33.01)
	Pain chest	**19.63 (0.22; 42.79)**	6.45 (− 15.45; 34.01)	2.84 (− 23.74; 38.68)	8.62 (− 16.56; 41.39)	20.01 (− 8.85; 57.99)
	Sputum	3.67 (− 20.83; 35.77)	7.94 (− 24.01; 53.31)	45.59 (− 7.68; 129.61)	24.46 (− 16.72; 86.02)	0.26 (− 34.07; 52.45)
	Anosmia/hypogeusia	− 1.56 (− 14.2; 12.94)	− 2.61 (− 18.55; 16.45)	3.32 (− 18.08; 30.31)	4.06 (− 15.21; 27.7)	22.74 (− 0.86; 51.96)
	Hospitalization	4.63 (− 25.82; 47.59)	8.15 (− 30.87; 69.19)	38.26 (− 22.67; 147.19)	31.32 (− 21.34; 119.23)	33.55 (− 21.74; 127.92)
	Oxygen	− 6.06 (− 37.76; 41.79)	24.27 (− 27.26; 112.31)	40.07 (− 30.12; 180.75)	22.2 (− 33.82; 125.64)	− 19.77 (− 57.68; 52.09)
	Cough	7.06 (− 7.33; 23.46)	3.06 (− 13.72; 25.31)	16.5 (− 8.05; 49.27)	14.89 (− 6.65; 43.13)	20.07 (− 3.52; 50.67)
	Obesity	− 15.41 (− 31.54; 4.53)	**− 25.48 (− 43.42; − 1.86)**	− 19.05 (− 43.38; 15.73)	− 19.68 (− 41.4; 10.09)	− 13.48 (− 37.72; 20.21)

Statistically significant variables indicated in bold font. *SARS-CoV-2 N* nucleocapsid, *FL* full length, *CT* C-terminus, *S* spike, *RBD* receptor-binding domain

Previous acute phase studies have shown that COVID-19 severity is associated with higher antibody responses. Here, hospitalization was associated with higher Ig levels many months after convalescence, suggesting that severity does not affect the stability of memory B cells and antibody-producing plasma cells [44–47]. Common symptoms such as fever and very specific symptoms such as altered smell and taste were also associated with higher antibody levels. Interestingly, hypertension was also positively associated with higher antibodies levels, consistent with some studies [29, 48] but contrary to others [49, 50]. We found that obesity was negatively related to IgM levels, similarly to post-vaccination studies in Italian HCW [50]. Smoking has been previously associated with lower antibody responses [28, 50–52], and we showed that this effect persists after several months, mainly affecting IgG. Finally, lower antibody levels in physicians and nurses in later time points could be due to work-related stress or burn out, which might affect immune memory fitness [53–55].

Limitations of this study include the lack of cellular or neutralizing antibody data, the specific focus on symptomatic HCW, and the limited sample size at later visits due to high vaccination coverage. Because of the screening of only those HCW with symptoms or contact with infected cases, we may have missed several reinfections. Another limitation is that we did not sequence the virus genome of the first infection and only some of the second infections; therefore, we cannot confirm reinfection with another SARS-CoV-2 variant. However, reinfections described occurred > 45 days after the first infection, and all of them had a negative RT-qPCR after the first infection and an increase in antibody levels after the 2nd infection. Future investigations should elucidate what threshold of antibodies correlate with protection against infection and disease, the determinants of antibody longevity, and what features of naturally-acquired antibody kinetics may predict that of vaccine-elicited responses.

In conclusion, our study demonstrates a robust persistence of SARS-CoV-2 antibodies after ~1.7 years, with seropositivity greater than 90% in unvaccinated individuals up to 20.5 months after COVID-19 symptoms onset. The maintenance of anti-S IgG, whose levels highly correlate with neutralizing antibodies [31], appears to be clinically relevant in protecting individuals particularly against the wild type and Alpha variants, despite lack of vaccination, consistent with having symptomatic reinfections in low responders and those reinfected with the more transmissible Delta variant. Antibody kinetics after natural infection appear to be stably sustained, more so than after vaccination, which has led to the implementation of booster immunizations, particularly in face of more contagious VoCs like Omicron. However, previously infected individuals also benefit from vaccination, as hybrid immunity seems to confer the greatest protection against SARS-CoV-2 infections and their symptoms [56].

## Supplementary Information

**Additional file 1: Table S1.** Association of baseline variables and comorbidities with levels of SARS-CoV-2 antibodies at time point 4 (January-February 2021) prior to the massive rollout of vaccination. **Table S2.** Seropositivity against the SARS-CoV-2 receptor-binding domain antigen from variants of concern. **Table S3.** Reinfections. **Table S4.** Significant associations of baseline variables with differential antibody rate of change. **Fig. S1.** Overall distribution of antibody responses for each isotype and antigen pair of the 128 pre-pandemic samples (negative controls) along with the cutoffs.

## Data Availability

Data and materials are available from the corresponding author upon request.

## Abbreviations

S: Spike
RBD: Receptor-binding domain
HCW: Health care workers
BSA: Bovine serum albumin
COPD: Chronic obstructive pulmonary disease
FL: Full length
PBS: Phosphate buffered saline
MFI: Median fluorescence intensity
PE: Phycoerythrin
RDTs: Rapid diagnostic tests
RT-qPCR: Reverse transcription quantitative polymerase chain reaction
T: Timepoint
VoC: Variants of concern
