# Supplementary material for: Sustained seropositivity up to 20.5 months after COVID-19
Source: BMC Med. 2022 Oct 13;20:379. doi: 10.1186/s12916-022-02570-3 (PMC9556137; doi:10.1186/s12916-022-02570-3)

**SUPPLEMENTARY MATERIAL**

# Sustained seropositivity up to 20.5 months after COVID-19

Carlota Dobaño^1,2^*, Anna Ramírez-Morros^3^*, Selena Alonso^1^, Rocío Rubio^1^, Gemma Ruiz-Olalla^1^, Josep Vidal-Alaball^3,4,5^, Dídac Macià^1,2^, Queralt Miró Catalina^3^, Marta Vidal^1^, Aina Fuster Casanovas^3^, Esther Prados de la Torre^1^, Diana Barrios^1^, Alfons Jiménez^1^, Jasmina Zanoncello^1^, Natalia Rodrigo Melero^6^, Carlo Carolis^6^, Luis Izquierdo^1,2^, Ruth Aguilar^1^, Gemma Moncunill^1,2#^, Anna Ruiz-Comellas^3,4,5,7#^

^1^ISGlobal, Hospital Clínic - Universitat de Barcelona, Barcelona, Catalonia, Spain

^2^CIBER de Enfermedades Infecciosas, Barcelona, Spain

^3^Unitat de Suport a la Recerca de la Catalunya Central, Fundació Institut Universitari per a la recerca a l’Atenció Primària de Salut Jordi Gol i Gurina, Sant Fruitós de Bages, Spain

^4^Grup de Promoció de la Salut en l’Àmbit Rural (ProSaARu), Institut Català de la Salut, Sant Fruitós de Bages, Spain.

^5^Facultat de Medicina, Universitat de Vic-Universitat Central de Catalunya (UVIC-UCC), Vic, Spain.

^6^Biomolecular screening and Protein Technologies Unit, Centre for Genomic Regulation (CRG), The Barcelona Institute of Science and Technology, Barcelona, Spain

^7^Centre d’Atenció Primària (CAP) Sant Joan de Vilatorrada. Gerència Territorial de la Catalunya Central, Institut Català de la Salut, Sant Fruitós de Bages, Spain

*^, #^ Shared authors

.

**ADDITIONAL FILE 1**

**Table S1.** Association of baseline variables and comorbidities with levels of SARS-CoV-2 antibodies at time point 4 (January-February 2021) prior to the massive rollout of vaccination. Only those variables that were selected in the stepwise procedure and were statistically significant in the multivariable regression models are shown.

^1^ Social worker, customer service, technician, driver, maintenance worker, IT worker, X-ray technician, others.

N, nucleocapsid; FL, full length; CT, C-terminus; S, spike; RBD, receptor-binding domain.

For IgM, the only significant variable was days since onset of COVID-19 symptoms, with the following transformed beta coefficients: RBD -0.31%, p<0.0001; S -0.26%, p<0.0001; S2 -0.25%, p<0.0001.

**Table S2**. Seropositivity against the SARS-CoV-2 receptor binding domain antigen from the different variants.

|  |  | **T0 (n=126)** | | **T6 (n= 72)** | | **T7 (n=39)** | | **T8 (n=23)** | |
| --- | --- | --- | --- | --- | --- | --- | --- | --- | --- |
|  | Positive | **n** | **%** | **n** | **%** | **n** | **%** | **n** | **%** |
| **IgA** | **Wuhan** | 82 | 64.57% | 32 | 44.44% | 14 | 35.90% | 17 | 73.91% |
|  | **Alpha** | 85 | 67.46% | 35 | 48.61% | 14 | 35.90% | 18 | 78.26% |
|  | **Beta** | 5 | 3.97% | 5 | 6.94% | 1 | 2.56% | 2 | 8.70% |
|  | **Gamma** | 36 | 28.57% | 7 | 9.72% | 3 | 7.69% | 10 | 43.48% |
|  | **Delta** | 88 | 69.84% | 29 | 40.28% | 9 | 23.08% | 18 | 78.26% |
| **IgG** | **Wuhan** | 118 | 92.91% | 64 | 88.89% | 35 | 89.74% | 22 | 95.65% |
|  | **Alpha** | 118 | 93.65% | 58 | 80.56% | 31 | 79.49% | 22 | 95.65% |
|  | **Beta** | 84 | 66.67% | 27 | 37.50% | 12 | 30.77% | 12 | 52.17% |
|  | **Gamma** | 111 | 88.10% | 46 | 63.89% | 25 | 64.10% | 19 | 82.61% |
|  | **Delta** | 118 | 93.65% | 50 | 69.44% | 28 | 71.79% | 22 | 95.65% |
| **IgM** | **Wuhan** | 70 | 55.12% | 11 | 15.28% | 3 | 7.69% | 11 | 47.83% |
|  | **Alpha** | 70 | 55.56% | 7 | 9.72% | 3 | 7.69% | 12 | 52.17% |
|  | **Beta** | 5 | 3.97% | 1 | 1.39% | 1 | 2.56% | 1 | 4.35% |
|  | **Gamma** | 13 | 10.32% | 1 | 1.39% | 1 | 2.56% | 2 | 8.70% |
|  | **Delta** | 75 | 59.52% | 10 | 13.89% | 4 | 10.26% | 15 | 65.22% |

T, timepoint. T0, July-August 2020. During early 2021 and before T6 (May-June 2021), Alpha was the predominant variant in Catalonia. Delta appeared in May in the study area and rose steadily till predominating (56-77%) in July 2021. During T7 (July 2021) and T8 (November 2021), Delta predominated (80-100%).

**Table S3**. Reinfections.

|  |  | **1^st^ episode** | | **2^nd^ episode** | |  |  |
| --- | --- | --- | --- | --- | --- | --- | --- |
| **Socio-demographic variables** | **Comorbidities** | **Symptoms period (level)** | **RT-qPCR results** | **Symptoms period (level)** | **RT-qPCR/RDT results** | **Period between infections** | **Serology** |
|  |  |  |  |  |  |  |  |
| Female, 59 yr, administrative | Smoking, hypertension, neurological disorder, depression, autoimmune disease | 23/03/20-  25/03/20  (mild) | 23/03/20 (+)  06/04/20 (-) | 20/05/20-22/05/20  First wave (mild) | 20/05/20 (+),  25/06/20 (-) | 1 month, 24 days  (58 days) | Not known 04/20  (+): 09/20 |
| Female, 42 yr, physician | None | 24/03/20-  25/05/20  (moderate) | 27/03/20 (+)  24/04/20 (-) | 08/20-10/20  Second wave  (mild -moderate) | 25/08/20 (+)  09/10/20 (-) | 4 months, 4 days  (130 days) | (-): 05 & 08/20  (+): 09/20 |
| Female, 30 yr, nurse | Obesity | 15/03/20-  14/05/20  (mild) | 02/04/20 (+)  22/04/20 (-) | 13/10/20-23/12/20  Second wave  (mild) | 13/10/20 (+) | 6 months, 25 days  (212 days) | (-): 09/20  (+): 10/20 |
| Female, 45 yr, physician | Autoimmune disease | 20/03/20- 03/04/20  (mild) | 25/03/20 (+),  06/04/20 (-) | No symptoms | 19/11/20 (+) | 7 months, 21 days  (239 days) | (+ weak): 09/20 |
| Male, 41 yr, nurse | None | 16/03/20-19/03/20  (mild) | 19/03/20 (+), 30/03/20 (-) | 07/12/20-09/12/20  Third wave  (mild) | 07/12/20 (RDT+)  22/12/20 (+) | 8 months, 18 days  (266 days) | (-): 09/20  (+): 07/21 |
| Female, 37 yr, nurse | Neurological disorder, obesity | 09/04/20-04/05/20  (mild) | 09/04/20 (+), 27/04/20 (-) | 09/01/21-22/01/21  Third wave  (mild) | 11/01/21 (RDT+), 21/01/21 (+) | 9 months,  (275 days) | (-): 10/20  (+): 07/21 |
| Female, 44 yr, nurse | None | 25/03/20-22/04/20  (mild) | 25/03/20 (+), 15/04/20 (-) | 09/07/21-22/07/21  Fifth wave  (mild) | 12/07/21 (+)  Delta B.1.617.2 | 1 year, 3 months, 11 days  (471 days) | (-): 03/21  (+): 07/21 |
| Female, 49 yr, nurse | None | 14/03/20-24/04/20  (mild) | 17/03/20 (+), 07/04/20 (-) | 21/10/21-05/11/21  Fifth wave  (mild) | 21/10/21 (+)  Delta B.1.617.2 | 1 year, 7 months, 7 days  (586 days) | (+): 07/21  (+): 10/21 |

**Table S4. Significant associations of baseline variables with differential antibody rate of change.** Transformed time-interaction beta values were obtained from regression coefficient estimates of interaction terms between baseline variables of interest and time since the onset of symptoms in linear mixed models as described in the Data Analysis section. We converted time since infection from days to months for an easier interpretation. Thus, transformed betas can be directly understood as antibody MFI percentual monthly increases (or decreases) over those antibody MFI values that would have been measured had no differential rate of change existed. P-values for each interaction term were obtained from t-statistics using Satterthwaite's method for denominator degrees of freedom [1]. Only significant interaction terms were reported in the table, out of 533 tests conducted for each baseline variable separately. Reported adjusted p-values were corrected for a false discovery rate of 5% following the Benjamini-Hochberg procedure [2].

1. Alexandra Kuznetsova, Per B. Brockhoff and Rune H. B. Christensen (2017) lmerTest Package: Tests in Linear Mixed Effects Models. *Journal of Statistical Software*, 82(13), 1–26. doi:10.18637/jss.v082.i13

2. Benjamini, Y., and Hochberg, Y. (1995). Controlling the false discovery rate: a practical and powerful approach to multiple testing. Journal of the Royal Statistical Society Series B, **57**, 289–300. <http://www.jstor.org/stable/2346101>.

**Figure S1.** Overall distribution of antibody responses for each isotype and antigen pair of the 128 pre-pandemic samples (negative controls) along with the seropositivity cutoffs.


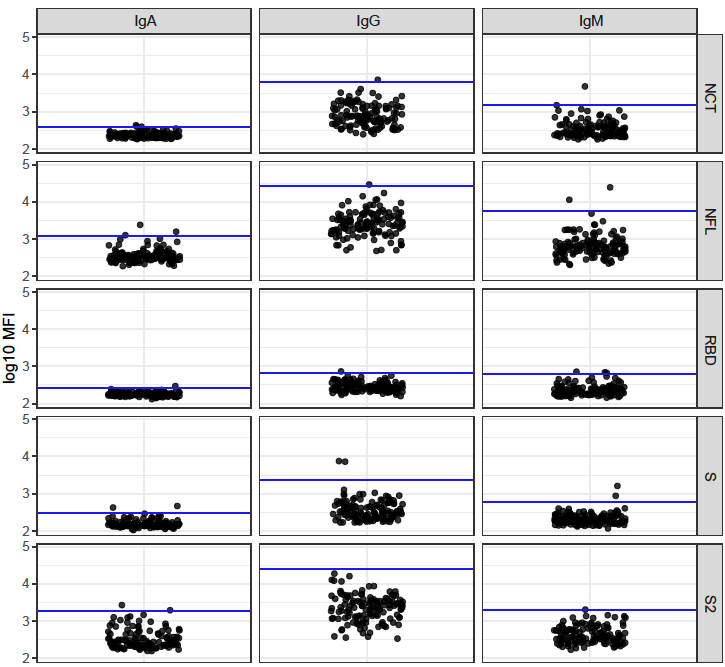

Supplement: Supplementary file 1 — Additional file 1: Table S1. Association of baseline variables and comorbidities with levels of SARS-CoV-2 antibodies at time point 4 (January-February 2021) prior to the massive rollout of vaccination. Table S2. Seropositivity against the SARS-CoV-2 receptor-binding domain antigen from variants of concern. Table S3. Reinfections. Table S4. Significant associations of baseline variables with differential antibody rate of change. Fig. S1. Overall distribution of antibody responses for each isotype and antigen pair of the 128 pre-pandemic samples (negative controls) along with the cutoffs. [file 12916_2022_2570_MOESM1_ESM.docx]
